# Comparison of flexible endoscopy and magnetic resonance imaging in determining the tumor height in rectal cancer

**DOI:** 10.1002/cnr2.1705

**Published:** 2022-08-17

**Authors:** Mohammed H. Basendowah, Mohammed A. Ezzat, Aseel H. Khayyat, Eyad Saleh A. Alamri, Turki A. Madani, Anas H. Alzahrani, Rana Y. Bokhary, Arwa O. Badeeb, Hussam A. Hijazi

**Affiliations:** ^1^ Department of Surgery, Faculty of Medicine King Abdulaziz University Jeddah Saudi Arabia; ^2^ Faculty of Medicine King Abdulaziz University Jeddah Saudi Arabia; ^3^ Department of Anatomical Pathology, Faculty of Medicine King Abdulaziz University Jeddah Saudi Arabia; ^4^ Radiology Department, Faculty of Medicine King Abdulaziz University Jeddah Saudi Arabia; ^5^ Radiation Oncology Unit, Radiology Department King Abdulaziz University Jeddah Saudi Arabia

**Keywords:** comparison, flexible endoscopy, histopathology, MRI, rectal cancer

## Abstract

**Background:**

Several modalities are available for the diagnosis of rectal cancer, including conventional gold standard rigid endoscopy and recent flexible endoscopy and magnetic resonance imaging (MRI). Each modality affects the management of these patients.

**Aim:**

To compare the accuracy of flexible endoscopy and MRI in the measurement of tumor height in patients with rectal cancer.

**Methods and Results:**

This study included 174 patients with rectal cancer who underwent flexible endoscopy and MRI for the measurement of tumor height. Data on patient demographics, comorbidities, treatment, and histopathology were identified and collected. We evaluate intraclass correlation coefficient (ICC) and Bland–Altman plot to test the agreement between the measurements. ICC were excellent with an ICC of 89% (95%CI 48%–99%). The mean ± standard deviation of the distance from the anal verge to the distal part of the tumor was 7.73 ± .47 for flexible endoscopy and 6.21 ± 0.39 for MRI, with mean difference of 1.52 (*p* ˂ .001). The accordance between the two modalities was not affected by sex, age, body mass index, histopathology, or metastasis.

**Conclusion:**

Excellent agreement between flexible endoscopy and MRI was noted, and no factor was found to affect such concordance.

## 1 INTRODUCTION

Rectal cancer is the leading cause of cancer‐related deaths worldwide.^1^, ^2^ It is considered highly prevalent in developed countries compared to its prevalence in less developed countries.^3^ In addition, it is more prevalent in men than in women.^4^ The tumor height of the rectum is defined as the distance between the anal verge and the distal end of the tumor.^5^ This height can be affected by numerous factors such as age, sex, tumor staging, luminal locations, anal sphincter tone, and patients' posture.^6^ The definitive cause of rectal cancer remains unknown; however, several risk factors are identified, such as high‐fat, low‐fiber diet; age >50 years, male sex, personal or family history, and predisposing conditions including hereditary nonpolyposis colorectal cancer (or Lynch), familial adenomatous polyposis, Gardner, Turcot, juvenile polyposis, Peutz–Jeghers syndromes, and inflammatory bowel disease.^7^, ^8^ There are different types of rectal cancer, the majority of which are adenocarcinomas (98%).^7^ Multiple modalities are available for the diagnosis of rectal cancer. Rigid endoscopy, which was considered the gold standard, but is no longer used in practice and has been replaced by flexible endoscopy. MRI^5^ and digital rectal examination are helpful modalities in the diagnosis.^9^ All of these modalities can affect and modify patient management.^10^ However, endoscopy (either rigid or flexible) and MRI have differences in determining the anatomical landmark of the rectum.^11^ In endoscopy, the landmarks include the dentate line and anal verge. The dentate line lies just beneath the anorectal junction and it is a scalloped demarcation formed by the anal valves, the beginning of the columns of Morgagni. The anal verge is the junction of hair‐bearing skin of the buttocks to non‐hair‐bearing skin of the anal canal and both are covered with squamous epithelium.^5^ Whereas in MRI, it is the anal verge or anorectal ring.^12^ The management of rectal cancer includes chemotherapy, radiotherapy, and surgery.^8^ It differs from patient to patient because it depends on identifying the location, size, and staging of cancer.^13^ A recent study involving 100 patients reported a small difference between MRI and flexible endoscopy (9.0 ± 23.5 mm) in measuring the tumor height and a larger difference between MRI and digital rectal examination (DRE) (40.9 ± 32.7 mm) and between DRE and colonoscopy (19.3 ± 17.3 mm).^6^ A study conducted in 2017 showed that tumor height was significantly lower when measured by MRI than when measured by flexible endoscopy, with a mean difference of 2.5 cm (95% CI [confidence interval]: 2.1–2.8).^5^ However, the measurement of tumor height differed between modalities, even in the same patient. Hence, this study aimed to compare the use of flexible endoscopy and MRI in the measurement of tumor height in patients with rectal cancer.

## 2 MATERIAL AND METHODS

### 2.1 Study design and participants

This is a retrospective observational study involving a sample of patients diagnosed with rectal cancer in Saudi Arabia, Jeddah. The inclusion criteria were a diagnosis of rectal cancer of any stage from 2017 to 2020, a flexible endoscopy report, an MRI report, age >18 years, and rectal cancer height ≤18 cm from the anal verge. The exclusion criterion was sigmoid cancer height >18 cm. This study primarily aimed to compare between flexible endoscopy and MRI in the diagnosis of rectal cancer by measuring tumor height. The tumor height was measured from the anal verge to the distal part of the tumor. Agreement between the measurements was tested using intraclass correlation coefficient (ICC), and ICC values ≥0.75 were considered as excellent agreement, 0.4–0.75 as fair to good agreement, and <0.4 as poor agreement.

### 2.2 Data collection

Using Google forms, two consultant surgeons collected and reviewed the patient data, including patients' demographics, BMI, and comorbidities such as DM, HTN, dyslipidemia, and hypothyroidism, as well as rectal cancer staging, management plan (type of surgery, chemotherapy cycles, radiotherapy doses), patient mortality, last follow‐up, MRI report, endoscopy report, and histopathology.

**FIGURE 1:**
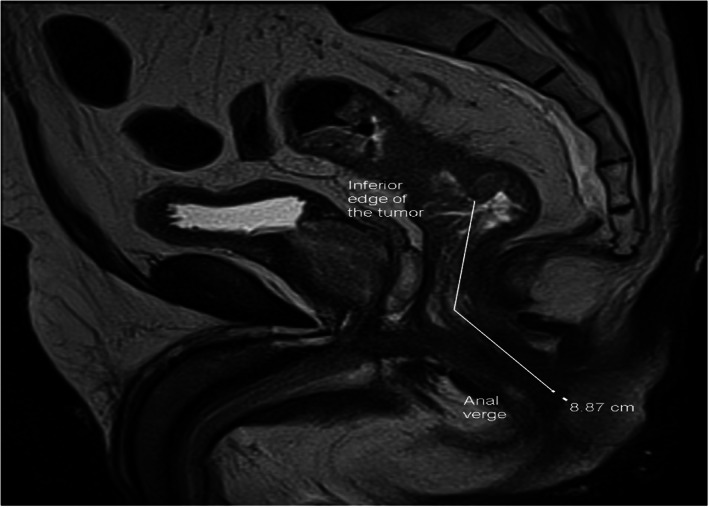
Sagittal T2 weighted images of a mid‐rectal circumferential tumor. Distance of the inferior edge of the tumor to the anal verge is shown by the white line measuring 8.8 cm. The measurement is obtained in the center of the anal and rectal lumen. The inferior edge of the tumor is shown by the white arrow and the anal verge is shown by the arrowhead

MRI images were obtained on a 1.5 Tesla Skyra Siemens MR magnet. The protocol includes multiplanar T1 and T2 weighted images of the pelvis as large field of views with pre and post contrast images as well as small field of view double oblique images of the rectal mass (perpendicular and parallel to the rectal tumor). Distances from the anal verge were obtained on the sagittal T2 weighted images or post contrast T1 weighted images (whichever showed the margins of the tumor the best). An expertized radiologist (co‐author AOB) obtained MRI images and measurement methods are shown in Figure 1.

### 2.3 Data analysis

Data were analyzed using SPSS version 26. We used one‐way random‐effects model to calculate the ICC. We used Python 3.2 to run Bland–Altman analysis and construct plots. We used mean and SD to summarized continues variables and frequency and percentage to summarized categorical variables. Two‐way *t*‐test were used to test the difference between MRI and endoscopy. *p*‐values <.05 were considered statistically significant. Ethical approval was obtained from the Research Ethics Committee Unit of King Abdulaziz University, Faculty of Medicine (ref no. 602‐20).

## 3 RESULTS

Patient demographics, comorbidities, type of surgery, histopathology, chemotherapy, and radiotherapy are shown in Table 1. A total of 174 patients were included in the study, of whom 92 (52.9%) were men, and 64 (36.8%) were Saudi Arabian. The mean ± SD of age was 58.67 ± 12.27, and that of BMI was 26.65 ± 5.97. Regarding comorbidities, 44 (25.3%), 52 (29.9%), 31 (17.8%), and 14 (8%) patients had diabetes mellitus, hypertension, peptic ulcer, and dyslipidemia, respectively. Fewer patients had chronic obstructive pulmonary disease 3 (1.7%), myocardial infarction 3 (1.7%), congestive heart failure 6 (3.4%), chronic liver disease 1 (0.6%), end‐stage renal disease 2 (1.1%), and hypothyroidism 7 (4%). Regarding the type of operation, 111 (63.8%) patients underwent surgery, with lower anterior resection being the most common surgery performed 55 (63.8%). Only 43 (24.7%) patients required intensive care unit admission, and 39 (22.4%) died. Histopathology showed that most patients had adenocarcinoma 162 (93.1%), whereas low‐grade dysplasia, malignant melanoma, and medullary carcinoma were the least common, with a prevalence of 0.6%. Most patients required chemotherapy 129 (74.1%), and more than half 102 (58.6%) required radiotherapy. The mean ± SD of the total dose of radiotherapy was 4624.80 ± 977.889 cGy and 227.16 ± 229.827 Gy. The mean ± SD of the fraction number of radiotherapy was 24.77 ± 7.45. The mean ± SD of dose per fraction of radiotherapy was 213.63 ± 91.02 cGy and 12.40 ± 19.26 Gy.

**TABLE 1:** Patient demographics, comorbidities, type of operation, histopathology, chemotherapy, and radiotherapy

Variables	
Male, *N* (%)	92 (52.9)
Saudi, *N* (%)	64 (36.8)
Age, Mean ± SD	58.67 ± 12.27
BMI, Mean ± SD	26.65 ± 5.97
Comorbidities: *N* (%)	
DM	44 (25.3)
HTN	52 (29.9)
Dyslipidemia	14 (8.0)
COPD	3 (1.7)
Peptic ulcer	31 (17.8)
MI	3 (1.7)
Congestive heart failure	6 (3.4)
Chronic liver disease	1 (0.6)
ESRD	2 (1.1)
Hypothyroidism	7 (4)
Metastasis, *N* (%)	28 (16.1)
Histopathology: *N* (%)	
Adenocarcinoma	162 (93.1)
Squamous cell carcinoma	4 (2.3)
High‐grade dysplasia	3 (1.7)
Low‐grade dysplasia	1 (0.6)
Neuroendocrine tumors	2 (1.1)
Malignant melanoma	1 (0.6)
Medullary carcinoma	1 (0.6)
Surgery, *N* (%)	111 (63.8)
Type of operation: *N* (%)	
Lower anterior resection	55 (63.8)
Abdominoperineal resection	19 (49.5)
Exploratory laparotomy	16 (17.1)
Colostomy	6 (18.0)
Total colectomy	2 (1.8)
Total pelvic exenteration	2 (1.8)
Total proctocolectomy	4 (1.8)
Laparoscopy	1 (3.6)
Unspecified	6 (0.9)
ICU admission, *N* (%)	43 (24.7)
Duration of ICU admission (in days), Mean (SD)	3.26 ± 3.9
Chemotherapy, *N* (%)	129 (74.1)
Radiotherapy, *N* (%)	102 (58.6)
Doses of radiotherapy, Mean ± SD	
Total dose of radiotherapy (cGy)	4624.80 ± 977.889
Total dose of radiotherapy (Gy)	227.16 ± 229.827
Number of fractions for radiotherapy	24.77 ± 7.45
Dose per fraction of radiotherapy (cGy)	213.63 ± 91.02
Dose per fraction of radiotherapy (Gy)	12.40 ± 19.26
Death	39 (22.4)

**FIGURE 2:**
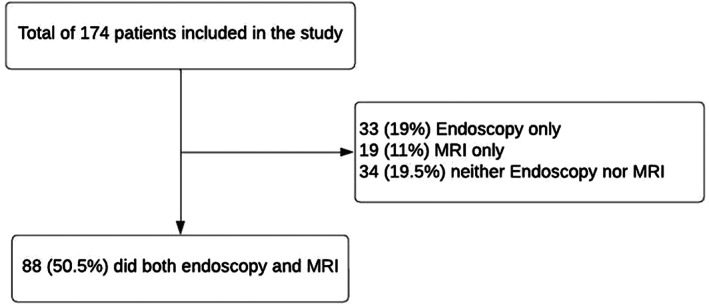
Shows the number of patients that underwent both endoscopy and MRI, endoscopy only, MRI only, and patient who did not do neither endoscopy nor MRI

**FIGURE 3:**
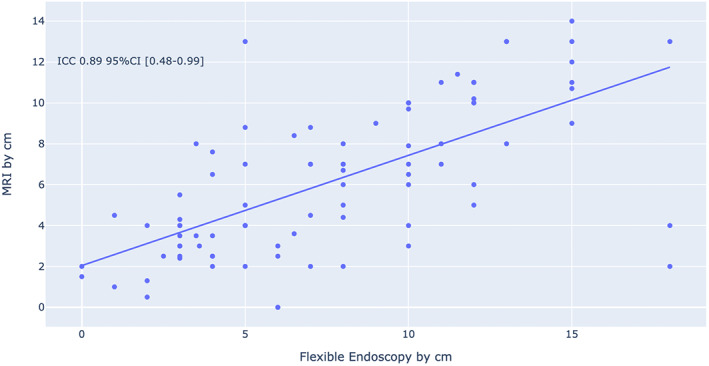
The agreement between rectal tumor height measured through MRI and endoscopy. Agreement between the measurements was tested using intraclass correlation coefficient (ICC)

**FIGURE 4:**
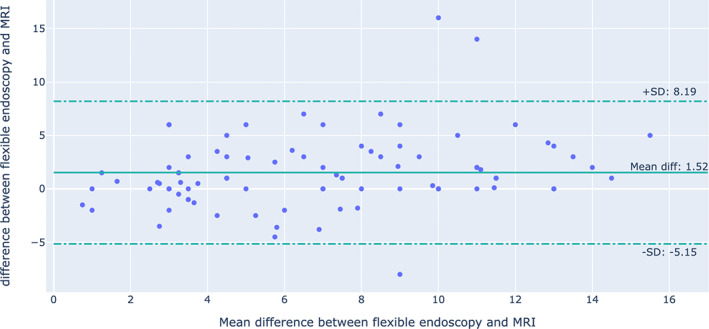
Bland Altman Plot to visualize the differences in measurements between MRI and endoscopy. The sold line represents the average difference in measurements. The dotted lines represent the upper and lower limits of the 95% confidence interval for the average difference

A comparison between the use of flexible endoscopy and MRI is shown in Table 2 and Figure 2. Of the 174 patients, 88 (50.5%) underwent both flexible endoscopy and MRI, 33 (19%) underwent flexible endoscopy only, and 19 (11%) underwent MRI only; the mean ± SD of the distance from the anal verge to the distal part of the tumor was 7.73 ± .47 for flexible endoscopy and 6.21 ± 0.39 for MRI, with mean difference of 1.52 (*p* ˂ .001). In Table 2, a significant difference was observed among men, metastasis, radiotherapy, histopathology, chemotherapy, and death between endoscopy and MRI. where the concordance of both tools was not affected by sex (*p* = .63), age (*p* = .89), BMI (*p* = .86), histopathology (*p* = .61), and metastasis (*p* = .79). Figure 3 shows the agreement between rectal tumor height measured through MRI and endoscopy was excellent at 89% (95%CI 48%–99%) Figure 4 shows Bland–Altman plot.

**TABLE 2:** Comparison between the use of flexible endoscopy and MRI in diagnosing rectal cancer

Variables	Endoscopy	MRI	* *p*‐value
Distance from the anal verge to the inferior edge of tumor, mean (SD)	7.73 ± .47	6.21 ± 0.39	<.001
Male sex, mean (SD)	8.16 ± 4.4	6.83 ± 3.75	.014
Metastasis, mean (SD)	8.11 ± 4.48	6.56 ± 3.77	.001
Radiotherapy, mean (SD)	7.45 ± 4.27	6.06 ± 3.6	.001
Adenocarcinoma, mean (SD)	8.11 ± 4.562	6.56 ± 3.923	<.001
Chemotherapy, mean (SD)	8.02 ± 4.29	6.43 ± 3.525	<.001
Death, mean (SD)	9.29 ± 5.64	4.93 ± 2.95	.031

* Two‐way *t*‐test were used to test the difference between MRI and endoscopy.

## 4 DISCUSSION

In this study, the accuracy of flexible endoscopy and MRI in the measurement of the tumor height in patients with rectal cancer was compared and excellent agreement was found between the two modalities. Rectal tumor height is the distance between the distal end of the tumor and the anal verge.^5^ Endoscopy is preferred for the diagnosis of rectal cancer, whereas pelvic MRI is preferred preoperatively for locoregional tumor staging.^8^, ^14^

In the current study, the patients' demographics, comorbidities, histopathology, and tumor data were investigated. The mean height was determined using both flexible endoscopy and MRI; a significant difference in the mean height between the two modalities was observed, although a uniform definition of the height was used. A significant difference in the distance measured by the two modalities was found (*p* ˂ .001). In addition, the two modalities were significantly affected by and varied between the male sex, metastasis, radiotherapy, histopathology, chemotherapy, and death. However, excellent agreement was noted between the two modalities, which was not affected by any factor, such as sex, age, BMI, histopathology, and metastasis. Although both modalities varied significantly in some variables, the agreement was not affected by any of the variables, and we assessed the variables showing excellent agreement.

The measurements of tumor height using various techniques may vary, which significantly leads to alterations in the treatment strategies and patient outcomes.^5^ Studies that determine the tumor height and diagnostic modality are lacking. Moreover, a uniform definition of rectal tumor height was not utilized in clinical trials. Therefore, in this study, we compared the use of flexible endoscopy and MRI in determining the tumor height using a uniform definition, that is, the distance from the anal verge to the distal part of the tumor.

In comparison to other studies, Jacobs et al. included 211 patients in their study; like our findings, they reported a significant difference in the tumor height, and the height assessed by MRI was lower than that assessed by endoscopy, with a mean difference of 2.5 cm. Similar to our findings, the agreement between the two modalities was excellent. The study suggested using MRI measurement for diagnostic purposes and treatment allocation due to its excellent inter‐ and intra‐observer agreement.^5^

A study by Attenberger et al. compared rectoscopy using three different MRI measurement techniques for rectal cancer height. The study revealed that MRI1 and MRI3 measures could be interchangeably used as a valid method to determine the tumor height compared to rigid rectoscopy, which was considered the gold standard.^15^ This may indicate that although flexible endoscopy is more recent and replaces conventional rigid rectoscopy, flexible endoscopy is still inferior to MRI. Previous studies that compared proctosigmoidoscopy with MRI‐defined tumor height revealed that measurements of both modalities differed significantly and were not interchangeable.^10^, ^11^, ^12^

The limitations of this study include the few comparisons with previous studies and a short discussion. We recommend doing the study in multi‐oncological center with high number of participants and with prospective approach, which includes correlation study of comorbidities, progression of disease, and death rate. The strengths of the study are as follows: this is the one of first study conducted on this subject and in Saudi Arabia and one of the fewest studies that investigated this research problem.

## 5 CONCLUSION

A higher tumor distance was revealed in flexible endoscopy findings than in MRI findings; however, excellent agreement between flexible endoscopy and MRI was noted, which was not affected by patients' demographics or metastasis.

## AUTHOR CONTRIBUTIONS

**Mohammed H. Basendowah:** Conceptualization (equal); methodology (equal); supervision (equal); writing – original draft (equal); writing – review and editing (equal). **Mohammed A. Ezzat:** Conceptualization (equal); data curation (equal); formal analysis (equal); funding acquisition (equal); investigation (equal); methodology (equal); project administration (equal); resources (equal); software (equal); visualization (equal); writing – original draft (equal); writing – review and editing (equal). **Aseel H. Khayyat:** Conceptualization (equal); methodology (equal); writing – original draft (equal); writing – review and editing (equal). **Eyad Saleh A. Alamri:** Conceptualization (equal); methodology (equal); writing – original draft (equal); writing – review and editing (equal). **Turki A. Madani:** Conceptualization (equal); methodology (equal); writing – original draft (equal); writing – review and editing (equal). **Rana Y. Bokhary:** Data curation (equal); project administration (equal); resources (equal); supervision (equal). **Arwa O. Badeeb:** Conceptualization (equal); data curation (equal); investigation (equal); project administration (equal); writing – review and editing (equal). **Hussam A. Hijazi:** Conceptualization (equal); methodology (equal); supervision (equal); writing – original draft (equal); writing – review and editing (equal).

## CONFLICT OF INTEREST

The authors have stated explicitly that there are no conflicts of interest in connection with this article.

## ETHICS STATEMENT

Ethical approval was obtained from the Research Ethics Committee Unit of King Abdulaziz University, Faculty of Medicine (ref no. 602‐20).

## Data Availability

The data will be provided upon request by corresponding author.
